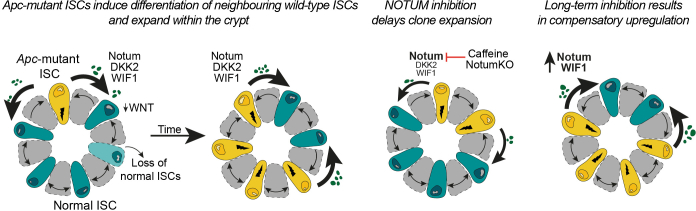# Caffeine Limits Expansion of *Apc*-Deficient Clones in the Intestine by NOTUM Inhibition

**DOI:** 10.1016/j.jcmgh.2023.06.008

**Published:** 2023-06-24

**Authors:** Milou S. van Driel, Jasmijn D.G. Linssen, Dustin J. Flanagan, Nikola Vlahov, Lisanne E. Nijman, Nina E. de Groot, Clara C. Elbers, Jan Koster, Owen J. Sansom, Louis Vermeulen, Sanne M. van Neerven

**Affiliations:** Amsterdam UMC, Location University of Amsterdam, Laboratory for Experimental Oncology and Radiobiology, Center for Experimental and Molecular Medicine, Amsterdam, The Netherlands; Cancer Center Amsterdam, Amsterdam, The Netherlands; Amsterdam Gastroenterology Endocrinology Metabolism, Amsterdam, The Netherlands; Oncode Institute, Amsterdam, The Netherlands; Amsterdam UMC, Location University of Amsterdam, Laboratory for Experimental Oncology and Radiobiology, Center for Experimental and Molecular Medicine, Amsterdam, The Netherlands; Cancer Center Amsterdam, Amsterdam, The Netherlands; Amsterdam Gastroenterology Endocrinology Metabolism, Amsterdam, The Netherlands; Oncode Institute, Amsterdam, The Netherlands; Amsterdam UMC, Location University of Amsterdam, Department of Gastroenterology and Hepatology, Amsterdam, The Netherlands; Department of Biochemistry and Molecular Biology, Monash University, Melbourne, Australia; Cancer Program, Biomedicine Discovery Institute, Monash University, Melbourne, Australia; Cancer Research UK Beatson Institute, Glasgow, United Kingdom; Cancer Research UK Beatson Institute, Glasgow, United Kingdom; Amsterdam UMC, Location University of Amsterdam, Laboratory for Experimental Oncology and Radiobiology, Center for Experimental and Molecular Medicine, Amsterdam, The Netherlands; Cancer Center Amsterdam, Amsterdam, The Netherlands; Amsterdam Gastroenterology Endocrinology Metabolism, Amsterdam, The Netherlands; Oncode Institute, Amsterdam, The Netherlands; Amsterdam UMC, Location University of Amsterdam, Laboratory for Experimental Oncology and Radiobiology, Center for Experimental and Molecular Medicine, Amsterdam, The Netherlands; Cancer Center Amsterdam, Amsterdam, The Netherlands; Amsterdam Gastroenterology Endocrinology Metabolism, Amsterdam, The Netherlands; Oncode Institute, Amsterdam, The Netherlands; Amsterdam UMC, Location University of Amsterdam, Laboratory for Experimental Oncology and Radiobiology, Center for Experimental and Molecular Medicine, Amsterdam, The Netherlands; Cancer Center Amsterdam, Amsterdam, The Netherlands; Amsterdam Gastroenterology Endocrinology Metabolism, Amsterdam, The Netherlands; Oncode Institute, Amsterdam, The Netherlands; Amsterdam UMC, Location University of Amsterdam, Laboratory for Experimental Oncology and Radiobiology, Center for Experimental and Molecular Medicine, Amsterdam, The Netherlands; Cancer Center Amsterdam, Amsterdam, The Netherlands; Cancer Research UK Beatson Institute, Glasgow, United Kingdom; Institute of Cancer Sciences, University of Glasgow, Glasgow, United Kingdom; Amsterdam UMC, Location University of Amsterdam, Laboratory for Experimental Oncology and Radiobiology, Center for Experimental and Molecular Medicine, Amsterdam, The Netherlands; Cancer Center Amsterdam, Amsterdam, The Netherlands; Amsterdam Gastroenterology Endocrinology Metabolism, Amsterdam, The Netherlands; Oncode Institute, Amsterdam, The Netherlands; Amsterdam UMC, Location University of Amsterdam, Laboratory for Experimental Oncology and Radiobiology, Center for Experimental and Molecular Medicine, Amsterdam, The Netherlands; Cancer Center Amsterdam, Amsterdam, The Netherlands; Amsterdam Gastroenterology Endocrinology Metabolism, Amsterdam, The Netherlands; Oncode Institute, Amsterdam, The Netherlands; Wellcome Trust–Cancer Research UK Gurdon Institute, University of Cambridge, Cambridge, United Kingdom

Colorectal cancer (CRC) development is characterized by stepwise accumulation of mutations, of which the majority display early mutations in tumor suppressor gene *APC.*^1^ Previous work revealed that *Apc* loss confers a competitive advantage to mutant intestinal stem cells (ISCs), which consequently replace all normal ISCs and drive crypt fixation in vivo.^2^ Recent studies demonstrate that this advantage can be attributed to the secretion of Wnt antagonists (eg, NOTUM/WIF1/DKK2) that drive normal ISC differentiation.^3^^,^^4^ In particular, NOTUM, which functions as negative regulator of Wnt activity by deacylating Wnt ligands,^5^ poses an interesting chemoprevention target because it is highly up-regulated in *Apc-*mutant murine and human cells.^3^^,^^4^^,^^6^ Interestingly, recent work has identified caffeine as a potent NOTUM inhibitor by binding its catalytic pocket and thereby inhibiting its function.^7^ Therefore, in this study, we investigate the chemopreventive effects of caffeine on the expansion of *Apc*-mutant clones in the intestine (see also Supplementary Methods).

To assess the role of caffeine on Wnt signaling in vitro, we first validated the recently described inhibitory effect of caffeine on NOTUM^7^ by using a Wnt reporter cell line (Supplementary Figure 1*A* and *B*). Administration of recombinant NOTUM decreased Wnt pathway activity, an effect that is alleviated by supplementing 200 μmol/L caffeine (Supplementary Figure 1*B*). Next, we investigated the effect of caffeine on intestinal organoids. We previously demonstrated that incubation of wild-type organoids with conditioned medium (CM) of *Apc*^-/-^ organoids resulted in loss of clonogenic potential.^3^^,^^4^ Moreover, we demonstrated that dilution of CM results in a dose-dependent rescue of clonogenicity, an effect that occurred at lower dilution using CM derived from *Apc*^-/-^;*Notum*^KO^ organoids,^3^ highlighting the importance of NOTUM in executing the inhibitory effect.^3^ Here, we reveal that addition of 200 μmol/L caffeine to *Apc*^-/-^ CM improves wild-type clonogenicity to a similar extent as previously reported using *Apc*^-/-^;*Notum*^KO^ CM^3^ (Figure 1*A*, Supplementary Figure 1*C* and *D*). Importantly, no effect was observed between organoids incubated with *Apc*^-/-^;*Notum*^KO^ CM with or without caffeine (Figure 1*A*, Supplementary Figure 1*E*). Together, these data indicate that caffeine reduces the Wnt inhibiting effects of *Apc*-mutant cells on their wild-type counterparts. Of note, caffeine treatment alone did not affect wild-type organoid growth, size, clonogenicity, and expression of Wnt target gene *Axin2* (Supplementary Figure 2*A–D*).

**Figure 1:**
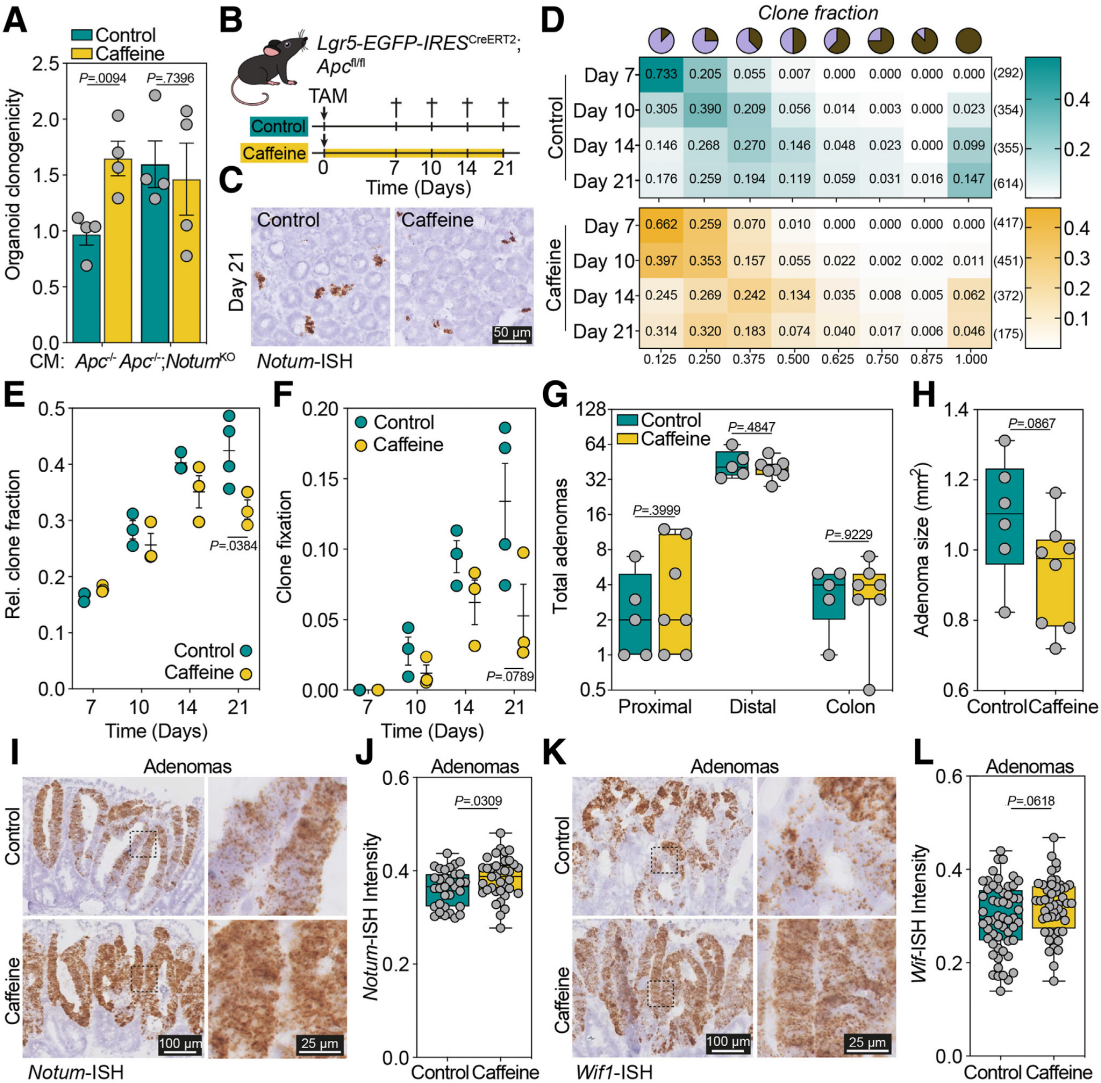
**(*A*) Clonogenicity of WT organoids incubated with 40% *Apc***^**-/-**^**CM or *Apc***^**-/-**^**;*Notum***^**KO**^**CM with/without caffeine (n = 4).** (*B*) Schematic illustration of in vivo experiment. (*C*) Detection of *Apc*-mutant clones using *Notum*-ISH. (*D–F*) Clone fraction distributions (n = no. of crypts) (*D*), average clone fraction (*E*), and crypt fixation (*F*) of *Apc*-mutant clones in the absence/presence of caffeine (n = 3–4 mice per group). (*G* and *H*) Adenoma number per intestinal region (*G*), average adenoma size in the distal SI (*H*) (n = 5–7 mice per group). (*I–L*) *Notum* (*I* and *J*) and *Wif1* (*K* and *L*) expression in adenomas of mice treated with/without caffeine (n = 3 mice, each dot is an ISH-positive region).

We next assessed the influence of caffeine on normal and mutant ISC dynamics in vivo. We induced *Apc* loss and traced the expansion of *Apc*-deficient clones over time in the absence or presence of caffeine administered in the drinking water (400 mg/L) (Figure 1*B*). As previously reported, *Apc*-mutant clones can be visualized by *Notum* expression (Figure 1*C*),^6^ and clone fraction distributions were followed over time (Figure 1*D*). We detected a significant reduction in the average clone fraction (Figure 1*E*) and observed a reduced number of fixed mutant clones (Figure 1*F*) in caffeine-treated mice. In line with our in vitro findings, wild-type ISC dynamics did not significantly change upon caffeine administration (Supplementary Figure 2*E–I*). Although the reduction in crypt fixation of *Apc*-mutant clones would suggest a subsequent reduction in adenoma development, long-term administration of caffeine does not impact the number and location of adenomas (Figure 1*G*). However, average adenoma size (Figure 1*H*) and the corresponding size distributions per animal (Supplementary Figure 3*A* and *B*) suggest that adenomas in caffeine-treated mice are generally smaller, pointing toward a delay in adenoma development. Closer analysis of these adenomas reveals a significant increase in *Notum* and elevated *Wif1* expression in response to long-term caffeine treatment (Figure 1*I–L*), whereas *Dkk2* expression is unaltered (Supplementary Figure 3*C* and *D*). These findings suggest that long-term NOTUM inhibition results in a compensatory up-regulation of Wnt antagonists. In line with this observation, long-term (21 days) in vitro caffeine administration to *Apc*-mutant organoids resulted in elevated *Notum* expression and significant *Wif1* up-regulation (Supplementary Figure 3*E–G*). Moreover, CM transfer of *Apc*-mutant organoids pretreated with caffeine for 21 days failed to rescue wild-type clonogenicity compared with CM of short-term (4 days) treated *Apc*-mutant organoids (Supplementary Figure 3*H*). To strengthen these findings, we analyzed adenomas from *Notum*^KO^ mice (Supplementary Figure 3*I*).^4^ Previous work revealed that concomitant *Apc* and *Notum* loss reduces adenoma size and number but does not completely prevent adenoma formation, suggesting that *Notum* loss also delays tumor initiation.^4^ To assess whether this is caused by compensatory up-regulation of Wnt antagonists, we quantified the expression of the 3 most up-regulated Wnt antagonists detected in this model^4^: *Notum*, *Wif1,* and *Dkk3*. As expected, we observed a marked decrease in *Notum* expression in *Notum*^KO^ mice (Supplementary Figure 3*J* and *K*). Furthermore, *Notum*^KO^ adenomas display increased *Wif1* expression (Supplementary Figure 3*L* and *M*), whereas *Dkk3* expression remains unchanged (Supplementary Figure 3*N* and *O*). Together, our findings reveal that long-term NOTUM inhibition by caffeine or conditional *Notum* loss activates a feedback loop that facilitates increased expression of Wnt antagonists resulting in progression of adenoma formation (Supplementary Figure 4). We specifically observe up-regulation of *Notum* and *Wif1*, which work at the Wnt-ligand level, but not *Dkk2* and *Dkk3*, which interfere with co-receptors LRP5/6, suggesting that this feedback loop could function to control the bioavailability of Wnt ligands. Our findings are especially relevant because of the vast global consumption of caffeinated beverages and the high prevalence of *APC* mutations in sporadic CRC. This study emphasizes the inherent difficulty of targeting the Wnt pathway as a cancer prevention strategy, and future research should consider the duration of caffeine administration as well as dosing schedules to avoid compensation. Although it remains unclear whether caffeine is the only putative cancer protective ingredient in coffee,^8^ our results could potentially explain why coffee intake is associated with a reduced risk of CRC development^9^ and progression.^10^

## Figures and Tables

**Supplementary Figure:**
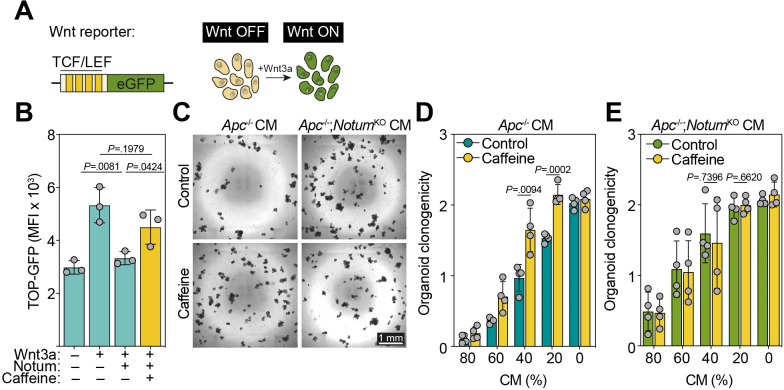


**Supplementary Figure:**
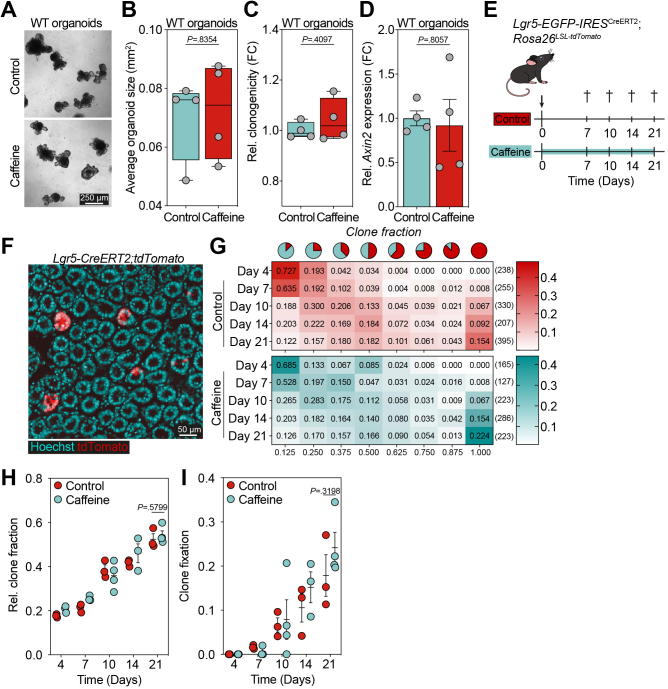


**Supplementary Figure:**
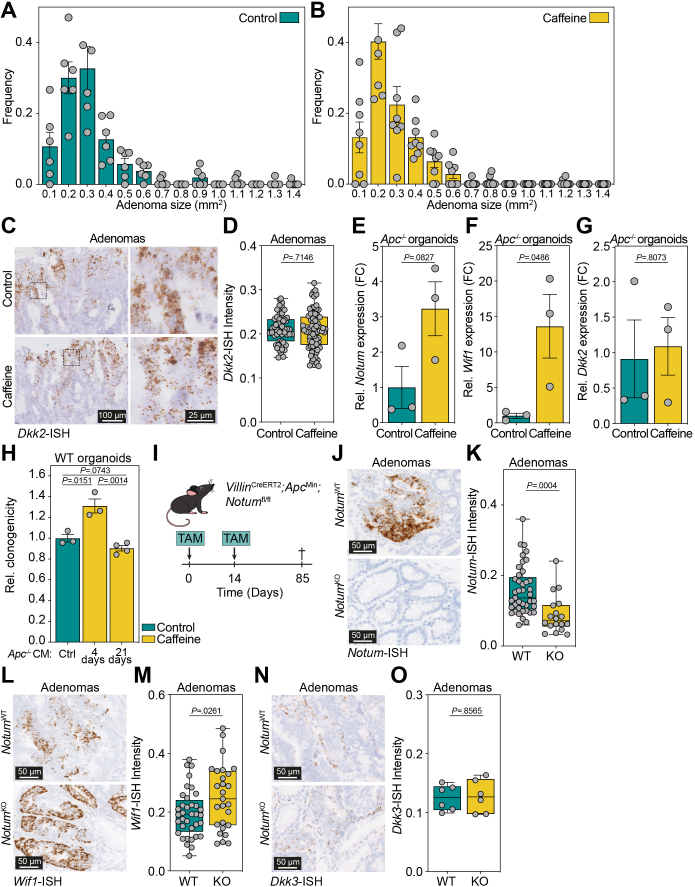


**Supplementary Figure:**