# Correction

**DOI:** 10.1016/j.jcmgh.2025.101471

**Published:** 2025-04-15

van Driel MS, Linssen JDG, Flanagan DJ, et al. Caffeine Limits Expansion of *Apc*-Deficient Clones in the Intestine by NOTUM Inhibition. Cell Mol Gastroenterol Hepatol 2023;16:652-655.

In the above article the figure legends for Supplementary Figures 1-4 are missing from the text. The supplementary figure legends are provided below.

**Supplementary Figure 1.***(A)* Schematic illustration of TOP-GFP Wnt reporter construct. *(B)* Wnt activation as measured by GFP expression (n=3). *(C-E)* Representative phase images of wells containing wild-type organoids that have been passaged after 5-day treatment of *Apc*-/- CM or *Apc*-/-;NotumKO CM in the absence or presence of caffeine *(C)*, and corresponding measurements of clonogenicity (*D-E*) (n=4).

**Supplementary Figure 2.***(A)* Phase images of wild-type organoids in the absence and presence of caffeine. (*B-D*) The size *(B)* relative clonogenicity *(C)* and relative *Axin2* expression *(D)* of wild-type organoids in the absence or presence of caffeine (n=4). *(E)* Schematic illustration of short-term experiment in *Lgr5-EGFP-IRES-CreERT2; Rosa26LSL-tdTom* mice in the absence or presence of caffeine. *(F)* Representative image of tdTomato+ clones 14 days after tamoxifen injection. **g**, Clone fraction distributions over time, (n=#crypts). *(H-I)* Relative clone fractions *(H)* and crypt fixation *(I)* of tdTomato+ clones in the absence or presence of caffeine. (n=3-4 mice per group).

**Supplementary Figure 3.***(A-B)* Size contributions of adenoma size in the distal small intestine of control mice (n=6 mice) *(A)* and caffeine treated mice (n=8 mice) *(B)*. *(C-D)* Representative images of *Dkk2* expression using *RNA*-ISH in adenomas of mouse treated with or without caffeine, *(C)*, and corresponding quantification of *Dkk2 (D)* intensity (n=3 mice, each dot is an ISH-positive region). *(E-G)*, Relative expression of Wnt antagonists *Notum (E)*, *Wif1 (F)*, and *Dkk2 (G)* in *Apc*-mutant organoids in the presence or absence of caffeine (n=3). *(H)* Relative clonogenicity of wild-type organoids cultured in *Apc*-*/-* CM without caffeine treatment (control) (n=3), or cultured in *Apc*-*/-* CM with caffeine treatment for either 4 days (short-term) (n=3) or 21 days (long-term) (n=4). *(I)* Schematic illustration of the experiment in *Villin*CreERT2;*Apc*Min;*Notum*fl/fl mice. *(J-O),* Representative images of *Notum (J)*, *Wif1 (L)* and *Dkk3* (N) expression using *RNA*-ISH in adenomas of *Villin*CreERT2;*Apc*Min;*Notum*fl/fl mice, and corresponding quantification of *Notum (K), Wif1 (M)* and *Dkk3 (O)* intensity (n=3 mice, each dot is an ISH-positive region).

**Supplementary Figure 4.** Schematic illustration depicting the proposed model of compensatory upregulation of Wnt antagonists secreted by *Apc*-mutant ISCs (*yellow*) upon long-term NOTUM inhibition. The bold arrows indicate *Apc*-mutant cells overtaking normal ISCs (*green, blue*) and the blunt-ended arrows indicate inhibition.

