# Supplementary material for: Caffeine Limits Expansion of Apc-Deficient Clones in the Intestine by NOTUM Inhibition
Source: Cell Mol Gastroenterol Hepatol. 2023 Jun 24;16(4):652–5. doi: 10.1016/j.jcmgh.2023.06.008 (PMC10511923; doi:10.1016/j.jcmgh.2023.06.008)
Supplement: Supplementary Methods [file mmc1.docx]

**Methods**

*Animal experiments*

*Lgr5-EGFP-IRES-Cre^ERT2^; Rosa26^LSL-tdTom^, Apc^fl/fl^* and *Villin*^CreERT2^;*Apc*^Min^;*Notum*^fl/fl^ models have previously been described^1–4^. All mice were maintained on a C57BL/6 background. *Rosa26^LSL-tdTom^* and *Apc^fl/fl^* animal experiments were performed according to national guidelines and approved by the Animal Experimentation Committee at the Amsterdam University Medical Centers (location Academic Medical Center) in Amsterdam (license number AVD1180020172125). *Apc*^Min^;*Notum*^fl/fl^ mouse experiments were performed in accordance with UK Home Office regulation (project license 70/8646) and adherence to the ARRIVE guidelines, and approved by the Animal Welfare and Ethical Review Board of the University of Glasgow. Animals were kept in regular housing conditions (12-hour light-dark cycle, between 19 °C-24 °C and 40-70% humidity) until sacrificed. At the start of all studies with *Rosa26^LSL-tdTom^* and *Apc^fl/fl^* models, mice were between 6-12 weeks old. Short-term experiments included both male and female mice, while long-term studies were performed using females. Animals were randomly assigned to control or caffeine treatments groups, of which sample sizes were determined based on previous studies^5,6^. Recombination was induced by an intraperitoneal (i.p.) injection of 0.2 mg (for *Rosa26^LSL-tdTom^* mice) or 2 mg (for *Apc^fl/fl^* mice) tamoxifen (Sigma) in sunflower oil. Caffeine (Sigma) treatment was orally administered by addition of caffeine (final concentration of 400 mg/L)^7,8^ in drinking water. Caffeine treatment started on the day of recombination initiation and continued until mice were sacrificed. In short-term experiments, mice were sacrificed at days 4, 7, 10, 14 and 21 after injection as this time span most accurately covers the process of labeling single cells until crypt fixation. In long-term studies, mice were sacrificed 60 days after injection as animals approached the humane end points due to adenoma development. At the start of studies with *Apc*^Min^;*Notum*^fl/fl^ mice, male and female mice were used between 6-8 weeks old. Mice received 2 mg i.p. injection tamoxifen per 25 mg body weight and were sacrificed 85 days after injection to isolate intestines for further processing.

*Tissue processing and clone fraction quantification*

Intestines were washed using ice-cold PBS and divided into proximal small intestine (SI), distal SI and colon prior to being cut longitudinally. In long-term studies, adenoma number and size were determined. Intestinal tissue was fixed overnight in the dark in 4% paraformaldehyde (PFA) solution followed by overnight preservation in 30% sucrose before tissue was frozen and stored (-80 °C). For clone fraction quantification, tissue slices (10 μm) were prepared using the Cryostat NX70 and placed on glass slips. To study the effect of caffeine on neutral drift dynamics (using *Rosa26^LSL-tdTom^*  mice), slides were counterstained with Hoechst-33342 and imaged on the SP8-X confocal microscope (Leica) using Leica Application Suite (LAS) software. To assess clone fraction distribution *Apc*-mutant clones, slides were stained for *Notum* mRNA and were counterstained with haematoxylin and imaged using the IntelliSite Ultra Fast 1.6 slide scanner (Philips). Clone fractions were quantified as proportions of the crypt circumference (in parts of eight: 1:8 to 8:8 (0.125-1.000)) and the distribution of clone fractions per timepoint was visualized in heatmaps. Crypt fixation is reached when a clone completely occupies a crypt (8:8 fractions).

*Organoid culture*

Mouse proximal SI crypts were isolated from wildtype and Lgr5;*Apc^fl/fl^* mice to generate organoids as previously described^9^. Organoid cultures were provided with medium consisting of advanced DMEM/F12 supplemented with 100X N2, 50x B27, 100X Glutamax, 5mM HEPES, 100X antibiotic/antimycotic (all Gibco), 1 mM *N-*acetyl-L-cysteine (Sigma), mouse EGF (50 ng/mL, TEBU-BIO), R-spondin1 and Noggin (both conditioned medium). *Apc*-mutant organoids were cultured in similar medium, but lacking R-spondin1. The first 2 days after crypt isolation, medium was supplemented with 5 μM CHIR99021 (Axon Medchem) and 10 μM ROCK inhibitor (Sigma). Cultures were maintained in a humidified incubator (37°C, 5% CO2). To recombine the *loxP*-flanked *Apc* alleles, organoids were incubated overnight with 1 μM 4OH-tamoxifen (Sigma) added to the medium. The next day, this was replaced for fresh medium. Efficiently recombined *Apc* organoids were selected by depletion of R-Spondin1 from the medium. To assess the ability of organoids to regrow after passaging, clonogenicity assays were performed. Organoids were cultured, subjected to different culture conditions and counted both before and after passaging. Duration of treatment before clonogenic potential was assessed is indicated in figure legends. For *in vitro* inhibition of NOTUM, caffeine (200 μM, Sigma) was administered to the medium immediately after plating organoids. For all experiments, medium was refreshed every other day. *Notum* knockout (KO) organoids used in this study were generated using CRISPR technology and described elsewhere^6^.

*Tissue culture*

Mouse embryonic fibroblasts (ATCC) containing WNT reporter TOP-GFP (35491, Addgene) were cultured in DMEM (Gibco) supplemented with 10% FCS, 1% glutamine, and antibiotic penicillin and streptomycin, and kept in a humidified incubator (37°C, 5% CO2). Cells were routinely checked for mycoplasma contamination. In TOP-GFP assays, cells were treated with WNT3A CM for 24 hours to stimulate GFP transcription. Similarly, cells were treated with caffeine (200 μM) and recombinant NOTUM (5μg/mL) for 24 hours. GFP positivity was measured by flow cytometry.

*Flow cytometry*

Flow cytometry was performed on the BD LSRFortessa (BD Biosciences) using FACSDiva software V8 (BD Biosciences). Data analysis was performed using FlowJo software.

*RNA extraction and RT-qPCR analysis*

To assess gene expression, SYBR Green (Roche) real-time qPCRs were performed using the LightCycler 480 system (Roche). RNA was extracted using the Nucleospin RNA isolation kit (#740955, Bioke). cDNA was generated using SuperScript III RT (Sigma). Gene expression levels were analyzed by applying the ΔΔCt method. All values were normalized to the expression of housekeeping genes *Hprt* and *Rpl37*. Primers used: *Axin2* forward 5’-CCATGACGGACAGTAGCGTA-3’, reverse 5’-CTGCGATGCATCTCTCTCTG-3’; *Notum* forward 5’-CTGCGTGGTACACTCAAGGA-3’, reverse 5’-CCGTCCAATAGCTCCGTATG-3’; *Wif1* forward 5’-CAAAGAATGCCAGCCATTCC-3’, reverse 5’-CAGCAAAGGGACATTGACAG-3’; *Dkk2* forward 5’-TCAGTCAGCCAACCGATCTG-3’, reverse 5’-TCTCTGTGGCATCGTTTCTTTT-3’; *Hprt* forward 5’-TGTAATGATCAGTCAACGGGGG-3’, reverse 5’-AGAGGTCCTTTTCACCAGCAA-3’; *Rpl37* forward 5’-CCAAGGCCTACCACCTTCAG-3’, reverse 5’-CAGTCCCGGTAGTGTTTCGT-3’.

*RNA-ISH*

To detect expression of *Notum* (probes #428981 and #472548), *Wif1* (probes #412361 and #412368), *Dkk3* (probe #400938) and *Dkk2* (probe #404841) mRNA, RNA-*ISH* was performed on FFPE and fixed-frozen tissue following manufacturers protocol (RNAscope 2.5 HD-Brown kit, ACD Bio). Staining intensity was measured using ImageJ software per adenoma.

*Statistics*Statistical analysis was performed by two-sided Student’s *t-test* unless indicated otherwise in the figure legend. *P*-values are displayed in the figures, threshold for significance α=0.05.

**Method references**

1. Barker N, et al*.* *Nature* 2007;499:1003–1007.

2. Kemp R, et al*.* *Nucleic Acids Res.* 2004;32:e92.

3. Shibata H, et al*.* *Science* 1997;278:120–133.

4. Flanagan DJ, et al*.* *Nature* 2021;594:430–435.

5. Vermeulen L, et al*.* *Science* 2013;342:995–998.

6. van Neerven SM, et al*.* *Nature* 2021;1–6.

7. Lu YP, et al*.* *Proc. Natl. Acad. Sci.* 2007;104:12936–12941.

8. Conney AH, et al. *Toxicol. Appl. Pharmacol.* 2007;224:209–213.

9. Sato T, et al. *Nature* 2009;459:262–265.
